# Diffuse Hyperpigmentierung bei systemischer Sklerose

**DOI:** 10.1007/s00105-025-05494-0

**Published:** 2025-03-26

**Authors:** Sven-Niklas Burmann, Johanna Matull, Frank Oellig, Jörg H. W. Distler, Alexander Kreuter, Andrea-Hermina Györfi

**Affiliations:** 1 Klinik für Dermatologie, Venerologie und Allergologie, Helios St. Elisabeth Klinik Oberhausen, Universität Witten/Herdecke, Josefstr. 3, 46045 Oberhausen, Deutschland; 2 Institut für Pathologie Mülheim an der Ruhr, Mülheim an der Ruhr, Deutschland; 3 Klinik für Rheumatologie, Medizinische Fakultät, Universitätsklinikum Düsseldorf, Heinrich-Heine-Universität Düsseldorf, Düsseldorf, Deutschland; 4 Hiller Forschungszentrum Rheumatologie, Medizinische Fakultät, Universitätsklinikum Düsseldorf, Heinrich-Heine-Universität Düsseldorf, Düsseldorf, Deutschland; 5 Klinik für Dermatologie, Venerologie und Allergologie, Helios St. Johannes Klinik Duisburg, Universität Witten/Herdecke, Duisburg, Deutschland

**Keywords:** Systemische Sklerodermie, Kuppennekrosen, Gefäßbeteiligung, Vaskularisation, Hautkolorit, Systemic scleroderma, Rat-bite necrosis, Vascular involvement, Vascularisation, Skin color

## Abstract

Pigmentstörungen sind ein häufig beobachtetes Phänomen bei Patienten mit systemischer Sklerose (SSc). Sie können bei bis zu 50 % der Betroffenen auftreten und umfassen sowohl Hypopigmentierungen als auch diffuse Hyperpigmentierungen. Letztere können ein Indikator für eine vaskuläre Beteiligung sowie hinweisend für einen schweren Verlauf der Erkrankung sein. Wir berichten über den Fall eines Patienten mit diffus kutaner SSc (dcSSc), bei dem neben schweren vaskulären Komplikationen, einschließlich digitaler Ulzerationen, Nekrosen und Autoamputationen, eine diffuse Hyperpigmentierung auftrat.

## Abstract (en)

Pigment disorders are commonly observed in patients with systemic sclerosis. They can occur in up to 50% of those affected and include both hypo- and diffuse hyperpigmentation. The latter can be an indicator of vascular involvement and suggest a severe course of the disease. We report on the case of a patient with diffuse cutaneous systemic sclerosis who, in addition to severe vascular complications including digital ulcerations, necrosis, and autoamputations, developed diffuse hyperpigmentation.

## Anamnese

**Abb. 1:**
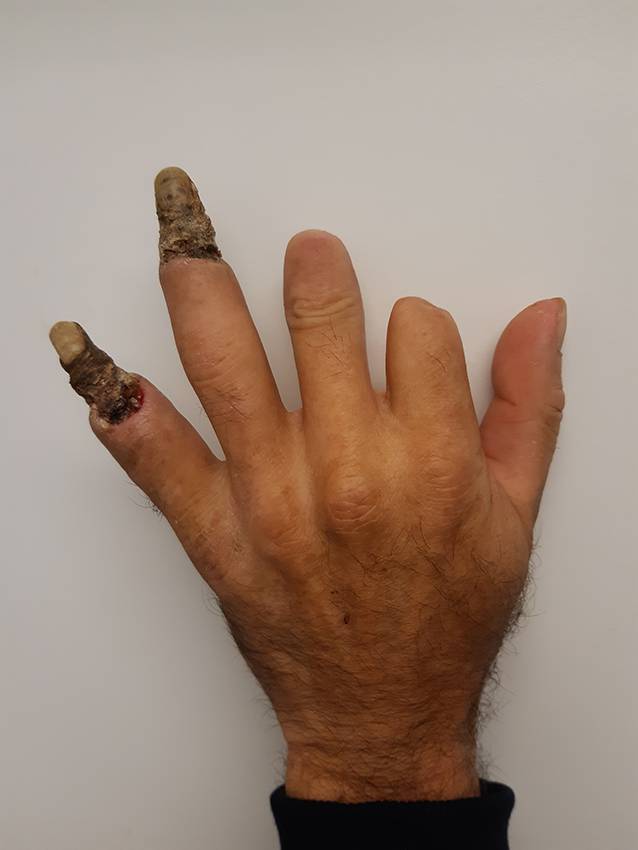
Übersichtsaufnahme der linken Hand. Im Bereich der Endphalangen der Digiti IV und V zeigen sich trockene Nekrosen. Zustand nach Amputation der Mittel- bzw. Endglieder der Digiti II und III der linken Hand

Ein 52-jähriger Patient stellte sich aufgrund einer schleichend progredienten Verhärtung der Finger, schmerzhaften Abblassens derselben sowie einer flächigen, diffusen Hyperpigmentierung des gesamten Integuments in unserer Klinik vor. Zum Vorstellungszeitpunkt bestanden zusätzlich trockene Nekrosen im Bereich der Endphalangen der Digiti IV und V der linken Hand (Abb. 1). In der Vergangenheit wurde in einer auswärtigen Klinik für Gefäßchirurgie nach Durchführung einer MR- und CT-Angiographie die Diagnose Thrombangiitis obliterans gestellt. Dort seien zudem Amputationen der Mittel- bzw. Endglieder der Digiti II und III der linken Hand sowie des Digitus II der rechten Hand durchgeführt worden. Zudem habe sich eine Autoamputation des Endglieds der linken Großzehe ereignet. Eine Raynaud-Symptomatik läge bereits seit einigen Jahren vor.

**Abb. 2:**
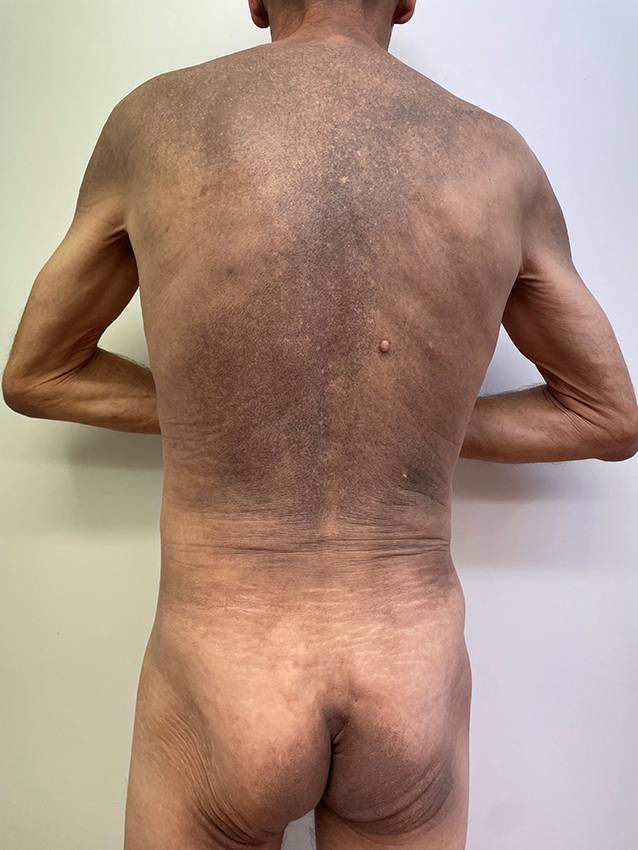
Dorsale Übersichtsaufnahme des Rumpfes. Flächige, schmutzig-graubraune bis schwärzliche Hyperpigmentierung des gesamten Rumpfes, der Glutäen sowie der proximalen Extremitäten neben vereinzelten, hypopigmentierten Makulae

**Abb. 3:**
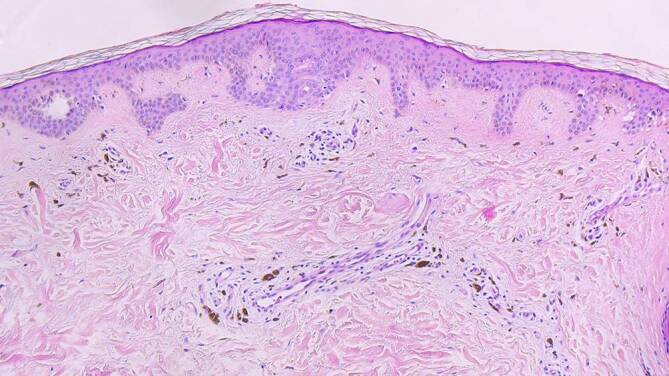
Mehrschichtig verhorntes Plattenepithel mit regelrechter korbgeflechtartiger Orthokeratose. Leichtgradige Hyperpapillomatose. Subepithelial reichlich Nachweis von Melanophagen in Nachbarschaft auch zu Hautanhangsgebilden. Eine Wandverdickung der Arteriolen ist nicht erkennbar. Hämatoxylin-Eosin-Färbung, Originalvergrößerung 100:1

Als relevante Nebendiagnose ließ sich eine periphere arterielle Verschlusskrankheit (pAVK) vom Schultertyp erheben mit vorausgegangener perkutaner Angioplastie und Stentimplantation der A. subclavia sinistra. Zudem berichtete der Patient auf Nachfrage über einen fortgesetzten Nikotinabusus (insgesamt 60 „pack years“).

Eine koronare Herzkrankheit sei in der Vergangenheit mit einer perkutanen Koronarintervention und nachfolgender dualer Thrombozytenaggregationshemmung mit Clopidogrel und Acetylsalicylsäure therapiert worden. Eine arterielle Hypertonie oder Dyslipoproteinämie bestand nicht.

## Hautbefund

In der körperlichen Untersuchung zeigte sich eine flächige, graubraune bis schwärzliche Hyperpigmentierung des gesamten Rumpfes, der Glutäen sowie der proximalen Extremitäten neben vereinzelten hypopigmentierten Makulae (Abb. 2). Im Bereich der Phalanges distales der Digiti IV und V der linken Hand imponierten trockene Nekrosen. Es zeigte sich weiterhin eine akral betonte Sklerose der Haut. In der Nagelfalzdermatoskopie ließen sich Megakapillaren und Einblutungen darstellen. Zentrofazial und palmar imponierten Teleangiektasien. Der modifizierte „Rodnan skin score“ betrug zum Zeitpunkt der Erstvorstellung 18 Punkte.

## Diagnostik

Serologisch konnten ein erhöhter Titer an antinukleären Antikörpern (ANA) von 1:320 sowie Antikörper gegen RNAse MRP (MRP = „mitochondrial RNA processing“), auch als Th/To-Antikörper bezeichnet, nachgewiesen werden. Eisenhaushalt, Kupfer- und Kortisol- sowie ACTH-Spiegel waren normwertig. Für eine pulmonalarterielle Hypertonie oder intrakardiale Thromben ergab sich echokardiographisch kein Hinweis. Die linksventrikuläre Ejektionsfraktion lag im Normbereich. Erhöhte Nierenretentionsparameter oder eine Proteinurie konnten wir nicht nachweisen. Wir führten zudem eine Tumorsuche mittels konventioneller Computertomographie und Positronenemissionstomographie durch; solide Tumoren ließen sich nicht darstellen. Gastroskopisch zeigte sich lediglich eine geringgradige, nicht aktive C‑Gastritis der Antrumschleimhaut. Eine Hautbiopsie der flächigen Hyperpigmentierung vom Rücken zeigte eine korbgeflechtartige Orthokeratose, leichtgradige Hyperpapillomatose sowie subepithelial reichlich Melanophagen (Abb. 3).

Entsprechend der aktuellen ACR-EULAR-Kriterien für die Diagnose und Klassifikation der systemischen Sklerose (SSc) wurde insgesamt eine Punktzahl von 12 erreicht (bei Patienten mit ≥ 9 Punkten gilt die Diagnose einer SSc als gesichert), wobei folgende Unterpunkte vorlangen: Sklerodaktylie (4 Punkte), digitale Ulzera (2 Punkte), Teleangiektasien (2 Punkte), abnormale Nagelpfalzkapillaren (2 Punkte), Raynaud-Phänomen (2 Punkte).

## Diagnose

Diffuse kutane SSc mit begleitender großflächiger Hyperpigmentierung.

## Therapie und Verlauf

Neben allgemeinen therapeutischen Maßnahmen wie einer prophylaktischen Hautpflege, Nikotinabstinenz, Bewegungstherapie, roborierenden Maßnahmen wie wechselwarmen Handbädern und Schutz vor Kälteeinwirkung erfolgte eine rheologische Therapie mit dem Prostazyklinanalogon Iloprost (Ilomedin®) über 5 Tage und einer regelmäßigen Wiederholung der Zyklen alle 8 Wochen. Zudem wurde eine Behandlung mit dem Endothelin-Rezeptorantagonist Bosentan ergänzt.

## Diskussion

Pigmentstörungen betreffen bis zu 50 % aller Patienten mit SSc [4]. Neben diffusen Hyperpigmentierungen wie im vorliegenden Fall können Hyperpigmentierungen in UV-exponierter Haut, perifollikuläre Hyperpigmentierungen („salt and pepper sign“), umschriebene, Vitiligo-ähnliche und diffuse Hypopigmentierungen sowie die Kombination von Hyper- und Hypopigmentierungen auftreten [5–7].

Insbesondere diffuse Hyperpigmentierungen erfordern eine sorgfältige endokrinologische Diagnostik zum Ausschluss von Adrenocorticotropin- und Melanozyten-stimulierendes Hormon produzierenden Tumoren. Auch Speicherkrankheiten mit Störungen des Kupferstoffwechsels (Morbus Wilson) oder des Eisenstoffwechsels (Hämochromatose) können zu Hyperpigmentierungen der Haut führen und sollten ausgeschlossen werden. Einer schiefergrauen Verfärbung der Haut kann eine Argyrose zugrunde liegen, bei der es zu Ablagerung von Silberkomplexen in der Haut kommt. Die Protonenpumpeninhibitor-induzierte Erythema dyschromicum perstans-ähnliche Hyperpigmentierung ist eine weitere Differenzialdiagnose. Sie tritt jedoch häufiger lokalisiert mit gräulichem Farbton auf und ist daher klinisch gut abzugrenzen, wenngleich histopathologische Ähnlichkeiten bestehen können [2].

Die genauen Ursachen für das Auftreten von Hyperpigmentierungen bei der SSc sind nicht vollständig verstanden. Diskutiert werden Mechanismen wie Entzündungsreaktionen, die zu einer vermehrten Melaninproduktion führen, eine verstärkte Sekretion von melanogenen Faktoren durch Endothelzellen, wie z. B. Endothelin‑1, oder Schädigungen der Haut, die eine verstärkte Pigmentablagerung zur Folge haben [1, 3].

Pigmentstörungen können – da sie häufig als kosmetisch störend wahrgenommen werden – erheblichen Einfluss auf die Lebensqualität der betroffenen Patienten nehmen. Neuere Untersuchungen deuten darüber hinaus auf eine Assoziation von diffusen Hyperpigmentierungen mit schwerem Verlauf einer SSc sowie vaskulärer Beteiligung wie pulmonalarterieller Hypertonie (PAH), renalen Krisen und digitalen Ulzerationen hin [3, 4, 6]. Dabei korreliert insbesondere das histopathologische Bild einer perivaskulären Hyperpigmentierung mit einem hohen Risiko für eine Gefäßbeteiligung [3].

Im Fall unseres Patienten bestand mit einer vorbekannten pAVK vom Schultertyp ein zusätzlicher aggravierender Faktor, der die Schwere der digitalen Nekrosen erklärt.

PAH und renale Beteiligung verlaufen in frühen Krankheitsstadien häufig asymptomatisch, stellen jedoch gleichzeitig potenziell lebensbedrohliche Komplikationen dar. Aus diesem Grund ist eine Identifizierung von Patienten mit erhöhtem Risiko für eine schwere vaskuläre Beteiligung bedeutsam, um eine sorgfältige Überwachung gewährleisten zu können. Pigmentstörungen können klinische Indikatoren für eine schwere vaskuläre Beteiligung darstellen. Betroffene Patienten sollten regelmäßig auf vaskuläre Komplikationen untersucht werden.

## Fazit für die Praxis

- Pigmentstörungen sind bei bis zu 50 % der Patienten mit SSc zu beobachten.
- Diagnose und Management von Pigmentstörungen bei SSc erfordern eine sorgfältige Untersuchung, um Erkrankungen wie Adrenocorticotropin-produzierende Tumoren und Stoffwechselstörungen auszuschließen.
- Angesichts der Assoziation von Pigmentstörungen und vaskulären Komplikationen wie digitalen Ulzerationen, pulmonalarterieller Hypertonie und renalen Krisen kann eine engmaschigere Überwachung von SSc-Patienten mit diffusen Hyperpigmentierungen in Erwägung gezogen werden.
